# Supplementary material for: The Efficacy of Molecular Analysis in the Diagnosis of Bone and Soft Tissue Sarcoma: A 15-Year Mono-Institutional Study
Source: Int J Mol Sci. 2022 Dec 30;24(1):632. doi: 10.3390/ijms24010632 (PMC9820733; doi:10.3390/ijms24010632)
Supplement: Supplementary file 1 [file ijms-24-00632-s001.zip › FIGURE S1.pdf]

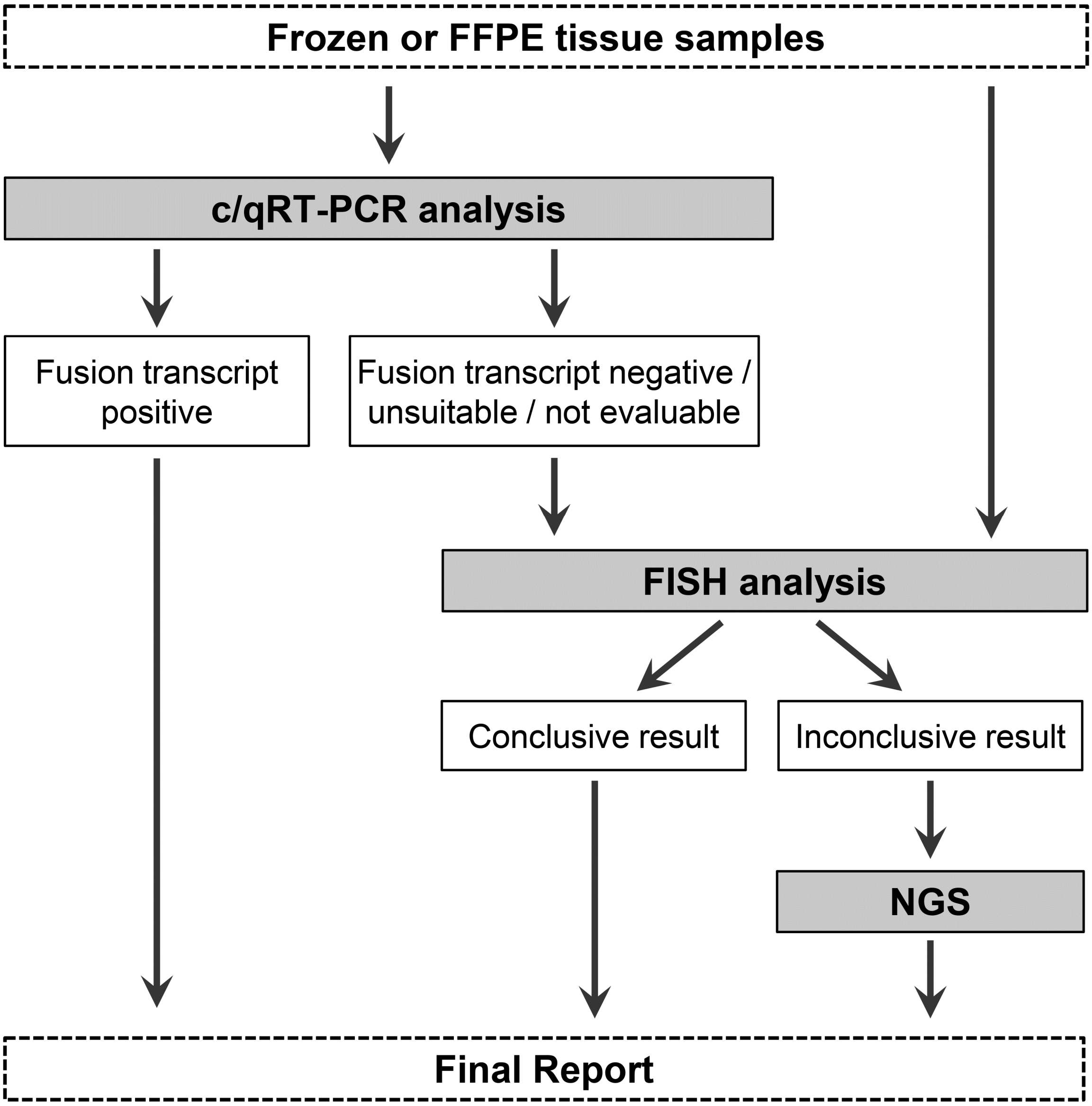

Figure S1 The algorithm shows the workflow used in our Institution to evaluate the presence of gene rearrangement on frozen or FFPE tissue samples.
