# Supplementary material for: The Efficacy of Molecular Analysis in the Diagnosis of Bone and Soft Tissue Sarcoma: A 15-Year Mono-Institutional Study
Source: Int J Mol Sci. 2022 Dec 30;24(1):632. doi: 10.3390/ijms24010632 (PMC9820733; doi:10.3390/ijms24010632)
Supplement: Supplementary file 1 [file ijms-24-00632-s001.zip › SUPP TABS1.pdf]

Supplementary Table S1 Molecular diagnostic tests available for bonde and soft tissue tumours at Rizzoli Institute

Tumor	Molecular alteration	Molecular method	Validated tissue	Source	Assay	Amplicon
**Alveolar Rhabdomyosarcoma**	t(1;13)PAX7-FKHR	qRT-PCR	FT/FFPET	B	Hostein-Cancer. 2004 Dec 15;101(12):2817-24. doi: 10.1002/cncr.20711.	<100bp
	t(2;13)PAX3-FKHR	qRT-PCR	FT/FFPET	A	Assay ID: Hs03024825_ft	98bp
**Alveolar Soft Part Sarcoma**	t(X;17)ASPL-TFE3	qRT-PCR	FT/FFPET	A	Assay ID:Hs03024642_ft Assay ID:Hs03024638_ft	<200bp
**Aneurysmal Bone Cyst ABC**	t(16;17)CDH11-USP6	RT-PCR	FT	LTD	CDH11-PE:TGGCGGCTCGGAGGTT USP6-PE:GCTCCTGTGCCTGCAAACTAT	>300bp
	t(16;17)CDH11-USP6	qRT-PCR	FFPET	A	Assay ID: Hs03024677_ft Assay ID: Hs03024675_ft	<110bp
	t(17;17)COL1A1-USP6	RT-PCR	FT	B	Panagopoulos-Cancer Genetics and Cytogenetics, 01 Jan 2008, 180(1):70-73<br>DOI: 10.1016/j.cancergencyto.2007.09.017	>300bp
**Angiomatoid Fibrous Histiocytoma**	t(2;22) EWS-CREB1	RT-PCR	FT/FFPET	B	Wang-Mod Pathol. 2009 Sep;22(9):1201-9. doi: 10.1038/modpathol.2009.85.	116bp
	t(12;22) EWS-ATF1	qRT-PCR	FT/FFPET	B	Coindre-Cancer. 2006 Sep 1;107(5):1055-64. doi: 10.1002/cncr.22099.	<100bp
**CIC-Rearranged Sarcoma**	t(4;19) e t(10;14) CIC-DUX4	qRT-PCR	FT	B	Machado-Virchows Arch (2013) 463:837–842 DOI 10.1007/s00428-013-1499-9<br>Gambarotti Histopathology. 2016 Oct;69(4):624-34<br>2006 KAWAMURA-SAITO HumMolGenet 15:2125-2137<br>2014 CHOI Am J Surg Pathol 2013;37:1379–1386	>200bp
	t(4;19) e t(10;14) CIC-DUX4	RT-PCR	FFPET	B	Classic PCR Gambarotti 2016, KAWAMURA 2006, CHOI 2014	<200bp
**Clear Cell Sarcoma**	t(12;22)EWS-ATF	qRT-PCR	FT/FFPET	B	Coindre-Cancer. 2006 Sep 1;107(5):1055-64. doi: 10.1002/cncr.22099.	<100bp
	t(12;22)EWS-ATF	RT-PCR	FT	B	Antonescu J Mol Diagn. 2002 Feb;4(1):44-52. doi: 10.1016/S1525-1578(10)60679-4.	>200bp
	t(2;22)EWS-CREB1	RT-PCR	FT/FFPET	B	Wang-Mod Pathol. 2009 Sep;22(9):1201-9. doi: 10.1038/modpathol.2009.85.	116bp
**Chondroblastoma**	H3F3B variant	RT-PCR	FT/FFPET	B	Girolami-J Clin Pathol2016;69:240–247. doi:10.1136/jclinpath-2015-203248	170bp
**Chondrosarcoma**	IDH1 and IDH2 variant	RT-PCR	FT/FFPET	B	Setty-J Mol Diagn. 2010 Nov; 12(6): 750–756. doi: 10.2353/jmoldx.2010.090237<br>Kerr-Am J Surg Pathol. 2013 Jun;37(6):787-95. doi: 10.1097/PAS.0b013e31827ab703.	120bp-170bp
**Dermatofibrosarcoma Protuberans**	t(17;22) COL1A1-PDGFB	qRT-PCR	FT	B	Szollosi-J Clin Pathol. 2007 Feb;60(2):190-4.doi: 10.1136/jcp.2006.037200. Epub 2006 May 26.	>200bp
**Desmoplastic Small Round Cell Tumor**	t(11;22) EWS-WT1	Classic PCR Murphy2008	FT/FFPET	B	Murphy-Hum Pathol. 2008 Dec;39(12):1763-70. doi: 10.1016/j.humpath.2008.04.019. Epub 2008 Aug 13.	>100bp
	t(11;22) EWS-WT1	qRT-PCR	FT/FFPET	LTD	EWS7A:GCAAGCTCCAAGTCAATATAGC<br>WT1-4:CGAGAAAACCTTCGTTCACAGTC<br>WTp:FAMAGCAGAGTGAGAAACCATACCAGTGTGACTTCAMGB	<200bp
**Epithelioid Haemangioendothelioma**	t(1;3)WWWTR1-CAMTA1	RT-PCR	FT	B	Errani-Genes Chromosomes Cancer. 2011 Aug;50(8):644-53. doi:10.1002/gcc.20886.	>200bp
	t(1;3)WWWTR1-CAMTA1	qRT-PCR	FFPET	LTD*	WWWTR1F2A:CCGGGCTGGGAGATGAC<br>CAMTA1ex8:CATTGCTGCAGGTCCACTTG<br>WWWTR1F2B:GGCTGGGAGATGACCTTCAC<br>CAMTA1ex9:TTTGGCGCTGTTACACTTGTG<br>WWWTR1 Probe:FAMCCACTGGCCAGAGGTMGB	<200bp
	t(X;11) YAP1-TFE3	RT-PCR	FT	B	Antonescu-Genes Chromosomes Cancer. 2013 Aug;52(8):775-84. doi: 10.1002/gcc.22073.	>200bp
**Epitheliod Haemangioma**	t(19;19) ZFP36-FOSB	qRT-PCR	FT/FFPET	LTD	ZFP36F2021:CAACCGTTACACCATGGATCTG<br>FOSBR2021:AGGTCCTGGCTGGTTGTGAT<br>ZFP36Probe2021:AAATGCCCGGTTCCT	<200bp
**Ewing sarcoma**	t(11;22) EWSR1-FLI1	RT-PCR	FT	B	Gamberi-JMolDiagn, 13(3) May2011. DOI:10.1016/j.jmoldx.2011.01.004<br>Lewis-Mod Pathol 2007 Mar;20(3):397-404. doi: 10.1038/modpathol.3800755.<br>Benini-CancerManagRes. 2018;10:doi: 10.2147/CMAR.S14162349–60.<br>Peter-Lab InvestActions. 2001 Jun;81(6):905-12.doi: 10.1038/labinvest.3780299 (BORDEAUX).	>300bp
	t(11;22) EWSR1-FLI1	RT-PCR	FT/FFPET	B	Lewis-ModPathol2007 +qPCRAlberti(Benini2018)	<200bp
	t(21;22) EWSR1-ERG	RT-PCR	FT	LTD-B	EWSB:ACCCACCCCAAACTGGATCC ERGB:CGAACTTGTAGGCGTAGCGC<br>Gamberi-JMolDiagn, 13(3) May2011. DOI:10.1016/j.jmoldx.2011.01.004<br>Lewis-Mod Pathol 2007 Mar;20(3):397-404.doi: 10.1038/modpathol.3800755.<br>Qian-DiagnMo Pathol 2005 Mar;14(1):23-8.doi:10.1097/01.pdm.0000140192.27878.97.<br>Benini-CancerManagRes. 2018;10:doi: 10.2147/CMAR.S14162349–60.<br>Peter-Lab InvestActions. 2001 Jun;81(6):905-12.doi: 10.1038/labinvest.3780299 (BORDEAUX).	>500bp
	t(21;22) EWSR1-ERG	RT-PCR	FT/FFPET	B	Classic PCR Lewis-ModPathol2007 e Qian-DiagnModPathol2005 +qPCRAlberti(Benini2018)	<200bp
	t(21;22) EWSR1-ERG	qRT-PCR	FT/FFPET	LTD	EWS10BS:CATGGATGAAGGACCAGATCTTG ERG10BS:TGACCGGTCCAGGCTGAT<br>EWS10:FAMTCTAGATTTACCATATGAGCCCMGB	62bp
	t(17;22) EWSR1-E1AF	RT-PCR	FT	B	Urano-Biochem Biophys Res Commun.1996 Feb 15;219(2):608-12. doi: 10.1006/bbrc.1996.0281.	410bp

**Ewing sarcoma**	t(7;22) EWSR1-ETV1	RT-PCR	FT/FFPET	B	Lewis-Mod Pathol.2007 Mar;20(3):397-404.doi: 10.1038/modpathol.3800755.	98bp
	t(17;22) EWSR1-ETV4	RT-PCR	FT/FFPET	B	Lewis-Mod Pathol.2007 Mar;20(3):397-404.doi: 10.1038/modpathol.3800755.	104bp
	t(4;22) EWSR1-SMARCA5	RT-PCR	FT	B	Sumegi-ModPathol 2011 Mar;24(3):333-42.doi: 10.1038/modpathol.2010.201.	>500bp
	t(6;22) EWSR1-POU5F1	RT-PCR	FT	B	Antonescu-Genes Chromosomes Cancer 2010 Dec;49(12):1114-24. doi: 10.1002/gcc.20819.	>300bp
	t(2;22) EWSR1-SP3	RT-PCR	FT	B	Wang-JournalMolDiagn, Vol.9, No.4Sep2007 DOI: 10.2353/jmoldx.2007.070053	<300bp
	t(2;22) EWSR1-FEV	RT-PCR	FT	B	Gamberi-JMolDiagn, 13(3) May2011. DOI:10.1016/j.jmoldx.2011.01.004Lewis-Mod Pathol	>300bp
	t(2;22) EWSR1-FEV	RT-PCR	FT/FFPET	B	Llombart-Bosch-DiagnMolPathol2000 Sep;9(3):137-44.doi: 10.1097/00019606-200009000-00003.	180bp
	t(16;21) FUS-ERG	RT-PCR	FT	LTD	FUSex5:gcagaaccagtacaacagca ERG2:gttgagcagctttcgactgg	404bp
	t(16;21) FUS-ERG	qRT-PCR	FT/FFPET	LTD	FUSex7:ggtggcttcaataatttgg ERG2:gttgagcagctttcgactgg ERGpe:FAMctcctggggggctcataMGB	84bp
	t(2;16) FUS-FEV	RT-PCR	FT	LTD	Gamberi-JMolDiagn, 13(3) May2011. DOI:10.1016/j.jmoldx.2011.01.004	>500bp
	t(2;16) FUS-FEV	RT-PCR	FT/FFPET	LTD	FUS:TGGAGGTAACTATGGCCAAGATC FEV:CAGCAGCTCCAGCAGAAACTG	172bp
**Exstraskeletal Mixoid Chondrosarcoma**	t(9;22)EWS-NR4A3	RT-PCR	FT	B	Benini-J Mol Diagn. 2014 May;16(3):314-23.doi: 10.1016/j.jmoldx.2013.12.002	>200bp
	t(9;22)EWS-NR4A3	qRT-PCR	FT/FFPET	B	Paioli and Benini-Ann Surg Oncol. 2021 Feb;28(2):1142-1150. doi: 10.1245/s10434-020-08737-7.	<200bp
	t(9;17)TAF2N-NR4A3	qRT-PCR	FT/FFPET	A	Assay ID: Hs03024453_ft	110bp
	t(9;15)TCF12-NR4A3	qRT-PCR	FT/FFPET	A	Assay ID: Hs03024487_ft	121bp
**Fibrous dysplasia**	GNAS variant	RT-PCR + Sanger	FT/FFPET	B	Fex8 TTCGGTTGGCTTTGGTGAG Rex8 GAGGGACTGGGGTGAATGTC<br>Fex9 TTGACATTCACCCCAGTCCC Rex9 AGAAGCAAAGCGTTCTTTACG	184bp<br>157bp
**Giant Cell Tumor of Bone**	H3F3A variant	RT-PCR	FT/FFPET	B	Girolami-J Clin Pathol. 2016 Mar;69(3):240-7. doi: 10.1136/jclinpath-2015-203248. Epub 2015 Sep 3.	239bp
	H3F3A variant	qRT-PCR	FT/FFPET	LTD	Gamberi-Pathol Res Pract. 2018 Jan;214(1):89-94. doi: 10.1016/j.prp.2017.10.023.	239bp
**Infantile Sarcoma**	t(12;15) ETV6-NTRK3	qRT-PCR	FT/FFPET	A	Assay ID: Hs03024415_ft	80bp
**Low Grade Fibromyxoid Sarcoma and Sclerosing Epitelioid Fibrosarcoma**	t(7;16)FUS-CREB3L2	RT-PCR	FT	B	Matsuyama-Am J Surg Pathol. 2006 Sep;30(9):1077-84. doi: 10.1097/01.pas.0000209830.24230.1f.	>300bp
	t(11;16)FUS-CREB3L1	RT-PCR	FT	B	Guillou-Am J Surg Pathol. 2007 Sep;31(9):1387-402. doi: 10.1097/PAS.0b013e3180321959.	>300bp
	t(7;22)EWS-CREB3L2	RT-PCR	FT	B	Lau-Am J Surg Pathol. 2013 May;37(5):734-8. doi: 10.1097/PAS.0b013e31827560f8.	>300bp
	t(11;22)EWS-CREB3L1	RT-PCR	FT	B	Lau-Am J Surg Pathol. 2013 May;37(5):734-8. doi: 10.1097/PAS.0b013e31827560f8.	>300bp
Mesenchimal Chondrosarcoma	t(8;8)HEY1-NCOA2	RT-PCR	FT	~~B~~	Low et al. BMC Cancer (2019) 19:182 doi.org/10.1186/s12885-019-5368-z	>200bp
	t(8;8)HEY1-NCOA2	RT-PCR	FT/FFPET	B	Andersson-Oncol Lett. 2014 Oct;8(4):1608-1612. doi: 10.3892/ol.2014.2364. Epub 2014 Jul 18.	119bp
**Mixoid Round Cell Liposarcoma**	t(12;16) FUS-DDIT3	qRT-PCR	FT/FFPET	A	Assay ID:Hs03024801_ft Assay ID:Hs03024813_ft	<200bp
	t(12;16) FUS-DDIT3	RT-PCR	FT	B	Antonescu-Clin Cancer Res.2001 Dec;7(12):3977-87. PMID: 11751490.	>200bp
	t(12;22)EWS-DDIT3	qRT-PCR	FT/FFPET	A	Assay ID:Hs03024858_ft	<100bp
	t(12;22)EWS-DDIT3	RT-PCR	FT	B	Antonescu-Clin Cancer Res. 2000 Jul;6(7):2788-93. PMID: 10914725.	>200bp
**Mioepitelioma**	t(1;22) EWS-PBX1	RT-PCR	FT	B	Brandal-GenesChromosomesCancer2008 Jul;47(7):558-64. doi: 10.1002/gcc.20559.	>500bp
	t(19;22) EWS-ZNF444	RT-PCR	FT	B	Brandal-GenesChromosomesCancer2009 Dec;48(12):1051-6. doi: 10.1002/gcc.20706.	>500bp
	t(6;22) EWSR1-POU5F1	RT-PCR	FT	B	Antonescu-Genes Chromosomes Cancer 2010 Dec;49(12):1114-24. doi: 10.1002/gcc.20819.	>300bp
**Pseudomiogenic Haemangioendothelioma**	t(7;19)Serpine1-FOSB	Classic PCR	FT	B	Walther-Journal of Pathology 2014, 232: 534-540. doi.org/10.1002/path.4322	>200bp
**Round cell sarcoma with EWSR1-non -ETS fusions**	EWSR1-NFATC2	RT-PCR	FT/FFPET	LTD	EWSex7PE:AGGAGGACGCGGTGGAAT<br>NFATC2ex3:CGATCCGCAGCTCGTAAG*	98bp
	t(1;22) EWSR1-PATZ1	Classic PCR Bridge2019	FT/FFPET	B	Bridge-Modern Pathology (2019)32:1593–1604https://doi.org/10.1038/s41379-019-0301-1 Mastrangelo-Oncogene 2000 Aug 3;19(33):3799-804. doi: 10.1038/sj.onc.1203762.	<200pb
**Sarcoma with BCOR-Genetic Alteration**	t(X;X)BCOR-CCNB3	qRT-PCR	FT/FFPET	LTD	BCOR Qbs:CAGTGATCTGGCCTCAGACAAC<br>CCNB3 Qbs:AATGAGTGGTTTCTCCATAATGTTT<br>CCNB3probe:CATAAGCTGGAAGTCACACCA	100bp
**Synovial Sarcoma**	t(X;18) SS18-SSX	qRT-PCR	FT/FFPET	A	Assay ID: Hs03024820_ft Assay ID: Hs03024398_ft	<100bp
**Solitary Fibrous Tumor**	inv(12)(q13q13) NAB2/STAT6	Classic PCR Robinson 2013 and Chmielecki2013	FT	B	Robinson-Nat Genet. 2013 Feb;45(2):180-5. doi: 10.1038/ng.2509. Epub 2013 Jan 13.<br>Chmielecki-Nat Genet. 2013 Feb; 45(2): 131–132. doi: 10.1038/ng.2522.	>200bp

Legend: A, Assay FAM-MGB (Assay LifeTechnologies); B, Bibliography source; LTD =Laboratory Developed test (custom home-made); qRT-PCR, Taqman Gense expression Assay; FT, frozen tissue; FFPET formalin-fixed paraffin embedded tissue
