# Supplementary material for: The Efficacy of Molecular Analysis in the Diagnosis of Bone and Soft Tissue Sarcoma: A 15-Year Mono-Institutional Study
Source: Int J Mol Sci. 2022 Dec 30;24(1):632. doi: 10.3390/ijms24010632 (PMC9820733; doi:10.3390/ijms24010632)
Supplement: Supplementary file 1 [file ijms-24-00632-s001.zip › SUPP TABS2.pdf]

Supplementary Table S2. FISH probes validated for bone and soft tissue tumours, available at the Rizzoli Institute

Probe name	LOCUS	Type	Manufacturer	Tumors involved
SPEC BCOR	(Xp11.4)	Dual Color BA Probe	Zytolight	Sarcoma with *BCOR* genetic alterations
SPEC CIC	(19q13.2)	Dual Color BA Probe	Zytolight	CIC-rearranged sarcoma
SPEC COL1A1	(17q21.33)	Dual Color BA Probe	Zytolight	Dermatofibrosarcoma protuberans
SPEC DDIT3	(12p13.3)	Dual Color BA Probe	Zytolight	Mixoid liposarcoma
SPEC ERG	(21q22.2)	Dual Color BA Probe	Zytolight	Ewing sarcoma
SPEC ETV6	12p13.2	Dual Color BA Probe	Zytolight	Infantile sarcoma
SPEC EWSR1	(22q12)	Dual Color BA Probe	Zytolight	Ewing sarcoma, Extrascheletal Myxoid Chondrosarcoma, Mixoid liposarcoma, Angiomatoid fibrous Histiocytoma, Low-grade fibromyxoid sarcoma, Desmoplastic small round cell tumor, Clear Cell Sarcoma, Myoepithelial tumour, Sclerosing Epitelioid Fibrosarcoma
FOS	14q24.3	Dual Color BA Probe	Empire Genomic	Epithelioid Haemangioma
SPEC FOXO1	(13q14)	Dual Color BA Probe	Zytolight	Alveolar rhabdomyosarcoma
SPEC FUS	(16p11)	Dual Color BA Probe	Zytolight	Ewing sarcoma, Mixoid liposarcoma, Angiomatoid fibrous Histiocytoma, Low-grade fibromyxoid sarcoma, Sclerosing Epitelioid Fibrosarcoma
SPEC -MDM2/CEN 12	(12q14.3-15)	Dual Color Probe	Zytolight	Well/dedifferentiated liposarcoma, Low-grade central osteosarcoma
NCOA2	8q13	Dual Color BA Probe	Empire Genomic	Mesenchimal Chondrosarcoma, Rhabdomyosarcomaa NCOA2-rarranged
SPEC NR4A3	(9q22.33)	Dual Color BA Probe	Zytolight	Extrascheletal Myxoid Chondrosarcoma
SPEC NTRK1	1q22-q23.1	Dual Color BA Probe	Zytolight	Many tumors
SPEC NTRK3	15q25.3	Dual Color BA Probe	Zytolight	Many tumors
SPEC PDGFB	(22q13,1)	Dual Color BA Probe	Zytolight	Dermatofibrosarcoma protuberans
SPEC SS18	(18q11.2)	Dual Color BA Probe	Zytolight	Synovial sarcoma
SPEC TFE3	(Xp11.23)	Dual Color BA Probe	Zytolight	Alveolar rhabdomyosarcoma, Epithelioid Haemangioendotelioma
SPEC USP6	(17p13.2)	Dual Color BA Probe	Zytolight	Aneurismal Bone Cyst, Ossifying Myositis, Nodular Fasciitis
SPEC WT1	(11p13)	Dual Color BA Probe	Zytolight	Desmoplastic small round cell tumor
SPEC WWTR1	(3q25.1)	Dual Color BA Probe	Zytolight	Epithelioid Haemangioendotelioma

Legend: BA, Brak apart
