# Supplementary material for: The Efficacy of Molecular Analysis in the Diagnosis of Bone and Soft Tissue Sarcoma: A 15-Year Mono-Institutional Study
Source: Int J Mol Sci. 2022 Dec 30;24(1):632. doi: 10.3390/ijms24010632 (PMC9820733; doi:10.3390/ijms24010632)
Supplement: Supplementary file 1 [file ijms-24-00632-s001.zip › SUPP TABS3.pdf]

Supplementary Table S3: Cases subjected to NGS analysis

Case	Preliminary diagnosis	Type of matherial	Preliminary negative c/qRT-PCR analysis	Preliminary negative FISH analysis	NGS results	Validation/orthogonal confirmation	Revised diagnosis
1	Malignant Peripheral Nerve Sheath Tumor (MPNST)	Frozen tissue	t(X;18)SS18-SSX, t(4;19)CIC-DUX4, inv(X)BCOR-CCNB3, t(12;16)FUS-DDIT3, t(12;22)EWS-DDIT3, t(8;8)HEY1-NCOA2	CIC, DDIT3, SS18	EWSR1/VEFZ1	Confirmed by qRT-PCR custom assay	Diagnostic confirmation
2	Undifferentiated round cell sarcoma	FFPE tissue	not required	not required	FAILED	n.a.	Diagnostic confirmation
3	Undifferentiated round cell sarcoma	FFPE tissue	t(12;16)FUS-DDIT3, t(12;22)EWS-DDIT3, t(8;8)HEY1-NCOA2, t(4;19)CIC-DUX4	EWSR1, CIC, DDIT3	No fuson detected	n.a.	Diagnostic confirmation
4	Ewing sarcoma	FFPE tissue	**Pos t(11;22)type1**	not required	EWSR1 -FLI1	Confirmed by qRT-PCR assay	Diagnostic confirmation
5	Malignant myoepithelioma	Frozen tissue	n.a.	Pos FISH EWSR1	EWSR1-PBX3	Confirmed by qRT-PCR custom assay	Diagnostic confirmation
6	Undifferentiated round cell sarcoma	FFPE tissue	t(X;18)SS18-SSX,t(12;22)EWSR1-ATF1, t(2;22)EWSR1-CREB1, EWS-NFATC2	SS18, EWSR1	No fuson detected	n.a.	Diagnostic confirmation
7	Undifferentiated Spindle cell sarcoma	FFPE tissue	t(12;15)ETV6-NTRK3	ETV6	No fuson detected	n.a.	Diagnostic confirmation
8	Undifferentiated spindle cell and epitheliomorphic sarcoma	Frozen tissue	t(9;22)EWSR1-NR4A3, t(9-15), t(9;17)TAF2N-NR4A3, t(4;19)CIC-DUX4	CIC, NR4A3	No fuson detected	n.a.	Diagnostic confirmation
9	Malignant myoepithelioma	FFPE tissue	t(X;18)SS18-SSX	SS18	No fuson detected	n.a.	Diagnostic confirmation
10	Undifferentiated round cell sarcoma	FFPE tissue	t(11;22)EWS-FLI1 , t(21;22)EWS-ERG, t(4;19)CIC-DUX4, t(20;22) EWS-NFATC2	DDIT3, NR4A3, CIC,	EWSR1-ATF1	Confirmed by qRT-PCR assay	Change of diagnosis (clear cell sarcoma with small cell areas)
11	Undifferentiated round cell sarcoma	FFPE tissue	t(X;18)SS18-SSX	SS18	No fuson detected	n.a.	Diagnostic confirmation
12	Undifferentiated spindle cell and epitheliomorphic sarcoma	Frozen tissue	t(12;16)FUS-DDIT3, t(12;22)EWS-DDIT3	DDIT3, MDM2	SS18-SSX1	Confirmed by qRT-PCR assay	Change of diagnosis (synovial sarcoma)
13	Malignant Peripheral Nerve Sheath Tumor (MPNST)	Frozen tissue	t(X;18)SS18-SSX, t(12;22)EWSR1-ATF1, t(2;22)EWSR1-CREB1	EWSR1	No fuson detected	n.a.	Diagnostic confirmation
14	Malignant Peripheral Nerve Sheath Tumor (MPNST)	Frozen tissue	not required	not required	No fuson detected	n.a.	Diagnostic confirmation
15	Ewing sarcoma	Frozen tissue	t(11;22)EWS-FLI1, t(21;22)EWS-ERG	**Pos EWSR1** (15% of nuclei) ERG, FUS	EWSR1-FEV	Confirmed by qRT-PCR assay	Diagnostic confirmation
16	Metastasis of malignant chondroblastoma	FFPE tissue	not required	not required	No fuson detected	n.a.	Diagnostic confirmation

Legend: PGM, Ion Personal Genome Machine; S5, Ion S5 next-generation sequencing; n.a., not applicable
